# Light-Mediated Contact Printing of Phosphorus Species onto Silicon Using Carbene-Based Molecular Layers

**DOI:** 10.1021/acs.langmuir.4c00763

**Published:** 2024-05-30

**Authors:** Patrick R. Raffaelle, George T. Wang, Alexander A. Shestopalov

**Affiliations:** † Department of Chemical Engineering, Hajim School of Engineering and Applied Sciences, University of Rochester, Rochester, New York 14627, United States; ‡ Sandia National Laboratories, Albuquerque, New Mexico 87185, United States

## Abstract

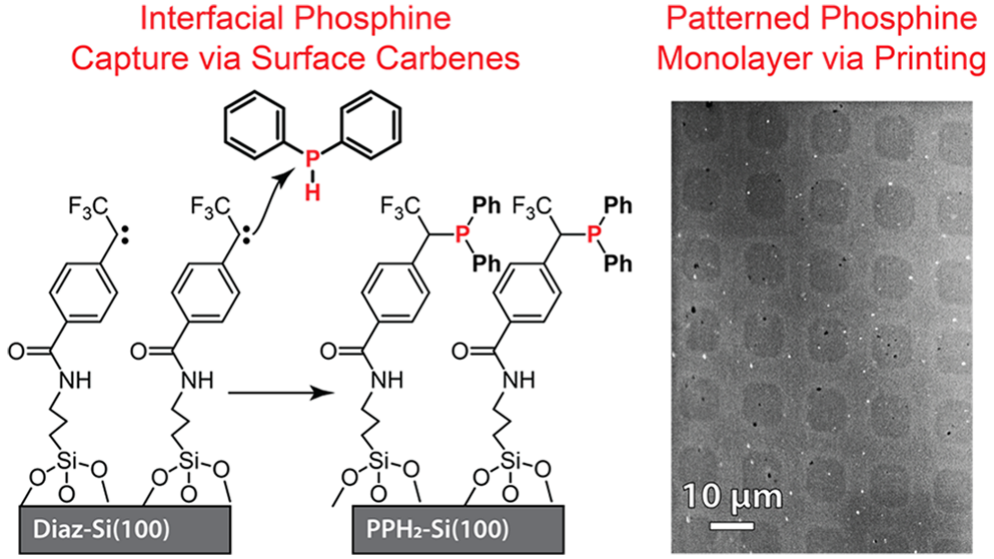

The ability to deposit pattern-specific molecular layers onto silicon with either regional p-/n-doping properties or that act as chemoselective resists for area-selective deposition is highly sought after in the bottom-up manufacturing of microelectronics. In this study, we demonstrate a simple protocol for the covalent attachment and patterning of a phosphorus-based dopant precursor onto silicon(100) functionalized with reactive carbene species. This method relies on selective surface reactions, which provide terminal functionalities that can be photochemically modified via ultraviolet-assisted contact printing between the carbene-functionalized substrate and an elastomeric stamp inked with the inorganic dopant precursor. X-ray photoelectron spectroscopy (XPS) analysis combined with scanning electron microscopy (SEM) imaging was used to characterize the molecule attachment and patterning ability of this technique. XPS spectra are indicative of the covalent bonding between phosphorus-containing molecules and the functionalized surface after both bulk solution-phase reaction and photochemical printing. SEM analysis of the corresponding printed features demonstrates the effective transfer of the phosphorus species in a patterned orientation matching that of the stamp pattern. This simple approach to patterning dopant precursors has the potential to inform the continued refinement of thin-film electronic, photonic, and quantum device manufacturing.

## Introduction

Strategies for the bottom-up manufacturing of semiconductor devices are increasingly relying on the selective functionalization of surfaces with small molecules or monatomic films that can act as deposition resists,^1,2^ dopants,^3^ or active device components.^4^ In combination with parallel patterning techniques, these molecular and atomic-scale interfaces can play an integral role in self-aligned fabrication schemes for biotemplates^5,6^ or doping of ultrashallow junctions^7,8^ or act as effective resists in area-selective deposition (ASD) techniques.^9−13^ However, traditional patterning methods, such as photolithography and shadow mask deposition, are not directly compatible with monomolecular or atomic layers, which are projected to play a bigger role in electronic device manufacturing as a result of the continuous downsizing of device components.^14^ Alternatively, contact transfer printing has been shown to be a viable bottom-up fabrication method for the patterning of molecules with up to 50 nm lateral resolution.^15,16^ This technique utilizes an elastomeric stamp inked with a molecule to transfer the species onto a chemically receptive receiver surface in a patterned orientation. Unlike traditional photolithographic techniques, photochemical transfer printing reactions are not always limited by light diffraction because the resolution is primarily determined by the diffusivity of the transferring molecules and the quality of the mechanical stamp–substrate contact.^17^ This technique is also naturally compatible with a wide range of materials, such as organics,^18^ inorganics,^19,20^ polymerics,^21^ and biologics.^22^ Furthermore, contact printing is inherently more efficient and less expensive than ultraviolet (UV)-based photolithographic techniques. Another advantage of transfer printing with elastic stamps is the ability to pattern nonplanar surfaces while still maintaining high feature uniformity and resolution,^23^ an impossible task for techniques such as photolithography^14,24^ and shadow mask deposition.^25,26^

When paired with a reactive interface, transfer printing can enable the delivery of monomolecular layers with regional p-/n-doping properties onto a semiconductor interface necessary for the development of ultrashallow doping strategies. Specifically, the immobilization of inorganic atoms, such as phosphorus and boron, to silicon is an essential component in the fabrication of ultrashallow doping interfaces for next-generation complementary metal–oxide–semiconductor (CMOS) transistors.^27,28^ To maintain low series resistance in sub-10 nm CMOS transistors, surface junctions should exhibit abrupt depth profiles and high phosphorus/boron concentrations that help to negate short-channel effects.^29−31^ Such properties cannot be achieved with current ion beam implantation techniques, which either produce broad, low-concentration dopant zones or inflict crystallographic damage upon the surface of a substrate over a micrometer range.^31−33^ Alternatively, plasma doping studies have demonstrated the ability to generate more conformal doping profiles;^34^ however, surface quality concerns arising from the entrapment of dopant molecules at the oxide interface during implantation have also been reported.^35^ Scanning tunneling microscopy (STM) has been shown to produce atomically precise phosphorus-doped regions on silicon using a STM probe to cleave Si–H bonds and generate strong and chemically inert Si–P bonds in a site-by-site manner.^8,36^ However, this deposition and patterning technique has very low throughput and requires ultrahigh vacuum conditions. Consequently, techniques such as transfer printing, which facilitate selective surface doping similar to that of STM but in parallel fashion and at milder conditions,^37^ are essential to circumvent the inherent throughput, material, and energy-intensive constraints of STM-based patterning.

To accomplish selective surface doping via contact printing, the receiver substrate needs to be reactive to the species being delivered via the transfer process. In this study, receiver substrates functionalized with a carbene-based molecular layer were examined on their ability to immobilize a printed phosphorus-based dopant precursor via stable C–P surface bonding. A carbene is a divalent carbon, which can be photogenerated from diazirine or diazo-based compounds. They can be generated from diazirines in either the solution or vapor phase and require a lower activation energy compared to most other organic radical-generating reactions.^38−40^ In our previous work, we have demonstrated the UV-activated insertion of carbene groups into Si–H surface bonds.^37,41^ However, carbene species can also provide a viable platform for chemically trapping organic and inorganic species at the solid interfaces from the gas or liquid phase via a similar UV-facilitated X–H insertion mechanism. Unlike other phosphorus-containing dopant precursors, which are typically immobilized using Si–O or C–O bonds, carbenes can directly insert into the P–H bond, creating strong and direct C–P attachment of phosphorus atoms.^42−44^ For example, diphenylphosphine (DPP) is a disubstituted phosphine derivative that can potentially be trapped by surface-immobilized carbene species via the insertion into the P–H bond and formation of the P–C bond. When coupled with the contact transfer printing, this would enable the selective immobilization of phosphorus-containing molecules via a direct C–P surface bonding.

In this study, a simple approach to selectively modify and pattern phosphorus-containing monolayers onto silicon(100) is demonstrated. Contact printing coupled with a carbene-based molecular system was utilized to introduce new surface functionality via conformal contact between functionalized silicon and a DPP inked elastomeric stamp. X-ray photoelectron spectroscopy (XPS) combined with scanning electron microscopy (SEM) and spectroscopic ellipsometry (SE) were used to characterize the phosphine attachment and patterning ability of this printing technique. This work has demonstrated (1) the direct trapping of phosphorus species by UV-generated surface carbenes immobilized to silicon and (2) the patterning of these species onto silicon with micrometer resolution and high feature uniformity, using contact printing under close-to-ambient conditions.

## Materials and Methods

All reagents and solvents were used as received without further purification. Solvents were purchased from Sigma-Aldrich and filtered through a 0.2 μm filter before use. The light-sensitive carbene precursor molecule 2,5-dioxopyrrolidin-1-yl 4-(3-(trifluoromethyl)-3*H*-diazirin-3-yl)benzoate (NHS-diazirine) was purchased from American Elements and stored in a dark environment. Its application was carried out under yellow light only. P-doped, monocrystalline (100) silicon substrates were purchased from University Wafer, Boston, MA, U.S.A. XPS spectra were recorded on a Kratos Axis Ultra XPS spectrometer equipped with an Al Kα (1486.6 eV) X-ray source at 200 W power and a pressure of 3.0 × 10^–8^ mbar. Survey scans were obtained between 0 and 1200 eV with a step size of 1 eV, a dwell time of 200 ms, and a pass energy of 140 eV averaged over 2 scans. Core-level region scans were obtained at the corresponding binding energy ranges with a step size of 0.1 eV, an average dwell time of 260 ms, and a passing energy of 20 eV averaged over 10 scans. Angle-resolved X-ray photoelectron spectroscopy (ARXPS) was used to collect P 2p spectra at varying emission angles to detect electrons from different surface depths (i.e., increasing the grazing angle limits detection to upper portion of the surface). XPS data were processed using CasaXPS software and instrument-specific atomic sensitivity factors. All C 1s peaks were calibrated to 284.7 eV, and this same binding energy shift was applied to all other spectra to account for adventitious carbon contamination. SEM images were recorded on a Zeiss Auriga focused ion beam scanning electron microscopy (FIB-SEM) microscope, detecting secondary electrons at ∼3.5 mm working distance. All goniometry analyses were gathered using ultrapure water. Ellipsometry data were collected using a J.A. Woollam M-2000 ellipsometer and fitted using the Cauchy refractive index model.

### Functionalization of the Si(100) Substrate with a Carbene Precursor

All glassware was washed with Nano-Strip solution (Cyantek) followed by rinsing with water and 99.5% isopropanol before being dried in an oven overnight at 130 °C. A 4 cm^2^ Si substrate was soaked in Nano-Strip for 5 min and then immersed in a 7:1 buffered oxide etch solution (12.5% hydrofluoric acid and 87.5% ammonium fluoride) for 30 s to chemically etch away a contaminated native oxide layer and then reimmersed in Nano-Strip for an additional 15 min at 70 °C to reform a clean oxide layer and to generate surface hydroxyl groups. The substrate was then rinsed with water and isopropanol and dried under nitrogen gas. The sample was then placed into a glass bottle containing 30 μL of 97% 3-aminopropyltrimetoxysilane (APTMS) purchased from Alfa Aesar and 5 μL of 99.5% triethylamine purchased from Sigma-Aldrich. The bottle was capped and heated to 65 °C and left for 2 h. After silanization, the sample was removed, rinsed thoroughly with dichloromethane and isopropanol, and then dried under nitrogen gas. The amino-terminated surface (NH_2_–Si) was subsequently immersed in a 10 mM solution of NHS-diazirine in 99.9% carbon tetrachloride for 2 h under yellow light. After the reaction, the diazirine-terminated surface (Diaz–Si) was rinsed with dichloromethane and isopropanol and then dried under nitrogen gas. The sample was stored in the dark under nitrogen until subsequent photochemical dopant species attachment.

### Light-Induced Generation of Reactive Carbene Species and Subsequent Covalent Attachment of the Phosphine Derivative

A Diaz–Si sample was placed into a UV-transparent vial and capped with a silicone septum. The vial was then purged for 5 min with argon before neat DPP was added dropwise to the surface of the sample via a needle syringe. The substrate was then put under an UV lamp (UVP UVGL-15, 365 nm, 4 W) for 1 h forming the DPP-reacted sample (PPh_2_–Si). Following the reaction, the sample was removed from the vial and rinsed with carbon tetrachloride and dried under nitrogen gas.

### Fabrication of the Micropatterned SiO_2_ Mold

NR9-1500PY (Futurrex) was spun on a clean silicon wafer at 3000 rpm for 40 s. The resulting substrate was baked on a digital hot plate at 155 °C for 2 min to produce 180 nm of the resist on Si. Photolithography (Karl Suss MA6/BA6) was performed using a photomask (Photo Sciences, Inc.) bearing 8 μm squares with an exposure time of 11.5 s. After UV exposure, the substrate was baked on a digital hot plate at 105 °C for 70 s, developed in RD6 (Futurrex) for 11 s, and immediately rinsed with water. The dried substrate was baked in an oven at 110 °C for 5 min and descumed in oxygen plasma for 1 min at 100 W and 6 × 10^–1^ mbar O_2_ pressure (Emitech K-1050X plasma asher). The oxide layer was etched away through the opening in the photoresist using reactive ion etching (Trion Technology Phantom II) for 22 min using CF_4_ and O_2_. Any remaining oxide was removed using buffered oxide etch (BOE). The negative resist was removed with Nano-Strip (at 55 °C for 2 min) producing a patterned silicon/SiO_2_ master.

### Preparation of Reactive Polyurethane Acrylate (PUA) Stamps

Synthesis of the PUA monomers were prepared according to a previously published protocol.^45^ Under yellow light, 1 mL of PUA was transferred into a vial and degassed at 30 inHg for 2 h. The resin was dispersed onto the patterned portion of the SiO_2_ mold and allowed to settle. An UV-transparent cover slide and 2.2 mm glass spacers were applied to control the stamp thickness. The mold was exposed to 365 nm UV light for 2 h. After removal of the cover slide, the replication system was again exposed to UV light overnight. The mold and resin were placed into an UV cross-linker system (Spectrolinker XL-1500, 351 nm, 6 × 15 W) for 600 s. After the system was heated on a hot plate to 65 °C for 10 min, the fully cured stamp was removed with a sharp-ended tweezer, rinsed with isopropanol, and dried under nitrogen.

### Generation of Micropatterned Phosphine Monolayers on the Functionalized Si Substrate via Contact Printing

A PUA stamp patterned with 8 μm squares was inked with a 50 μL droplet of neat DPP, allowed to dry in ambient air for 10 min, and then thoroughly dried under nitrogen gas. The stamp was placed on the top of a diazirine-terminated Si substrate at room temperature for 5 min with no external load. The stamp/substrate system was exposed to 351 nm UV light for 2 min in the UV cross-linker system. After the reaction, the substrate was carefully removed from the stamp, rinsed with isopropanol, and dried under nitrogen gas.

## Results and Discussion

### Immobilization and Activation of Carbene for Subsequent Capture of Phosphorus Species

**Figure 1:**
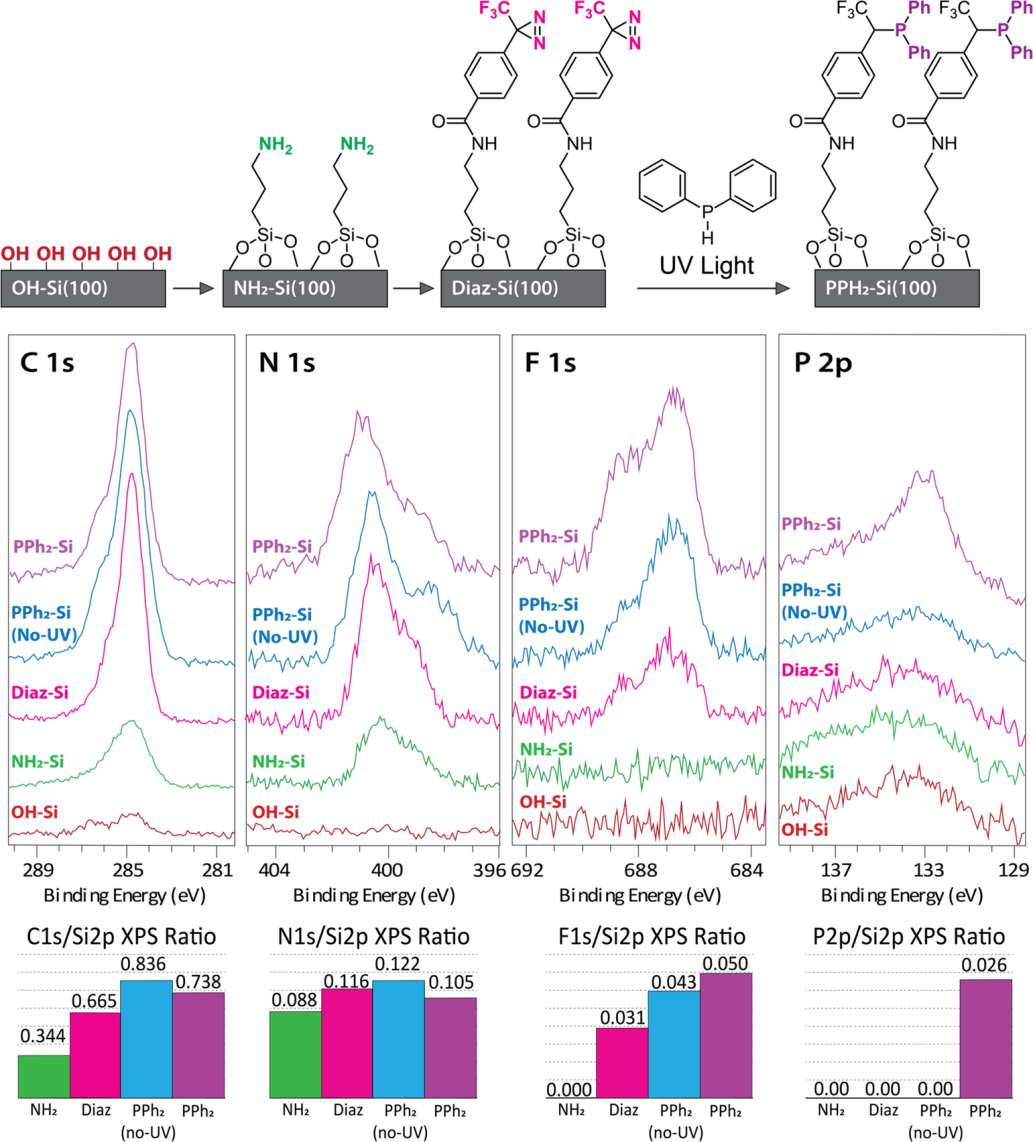
(Top) Illustration of carbene-based functionalization, (middle) C 1s, N 1s, F 1s, and P 2p XPS region spectra measured from the following surfaces: NH_2_–Si, Diaz–Si, PPh_2_–Si (no UV), and PPh_2_–Si represented by the green, pink, blue, and purple profiles, respectively, and (bottom) histograms showing the quantitative XPS characterization of region scans (C 1s, N 1s, F 1s, and Si 2p) for each surface, all normalized by the Si 2p peak intensity.

Transfer printing requires a receiver substrate chemically receptive to the species being delivered during the transfer step. In this study, we evaluated the use of surface-immobilized carbenes as reactive species that can trap substituted monophosphines via direct insertion into P–H bonds. To immobilize unreacted carbene precursors onto Si, heterobifunctional molecules that consist of a carbene precursor species and surface-reactive functional groups are required. As shown in the reaction scheme in Figure 1, we theorized that a NHS-diazirine molecule could react with a primary amine species via the *N*-hydroxysuccinimide constituent at one end of the molecule, while the diazirine headgroup can be independently activated under UV light to generate carbenes. The functionalization strategy employed in this study relied on a bilayered system, consisting of a primary aminosilane layer attached to a secondary diazirine layer via stable amide bonding. Organosilanes have been shown to form homogeneously oriented monolayers on oxidized group IV semiconductors^46^ and have good chemical and physical stability. From the diazirine precursor, metastable^47^ surface carbenes were generated following exposure to UV light at 365 nm. The dense primary silane layer increased the chemical and physical stability of the substrate interface, while the secondary reactive overlayer provided a functional surface moiety that could directly trap P atoms via stable covalent C–P bonding without any additional linkers. The phenyl rings present in the immobilized DPP species can potentially be dissociated either photochemically or through low-temperature annealing.^48,49^ XPS, SE thickness, and contact angle measurements were collected to characterize the incorporation of each functionalization step and are presented in Figure 1 and Table 1.

**Table 1:** Water Contact Angle and Ellipsometry Measurements Performed on NH_2_–Si, Diaz–Si, PPh_2_–Si, and PPh_2_–Si (no UV) Substrates

	ellipsometry		goniometry	
surface	layer thickness (nm)	theoretical thickness (nm)	contact angle (deg)	hysteresis (deg)
NH_2_–Si	0.98 ± 0.06	0.83	60.0 ± 0.1	17.0 ± 0.1
Diaz–Si	1.34 ± 0.06	1.31	68.0 ± 0.1	25.0 ± 0.1
PPh_2_–Si	1.62 ± 0.06	1.37	55.0 ± 0.1	34.0 ± 0.1
PPh_2_–Si (no UV)	1.43 ± 0.06	1.31	64.0 ± 0.1	31.0 ± 0.1

Figure 1 shows the C 1s, N 1s, F 1s, and P 2p core-level XPS spectra of hydroxyl-terminated silicon (OH–Si) following silanization, carbene activation, and dopant attachment steps. A fourth control sample was also included to examine the necessity of UV exposure for carbene generation and dopant capture. The primary contribution to each of the C 1s spectra was from C–C/C=C bonds centered around 284.7 eV. Following silanization, there was a shift toward 286 eV, which is indicative of the chemical contributions from C–N bonding arising from the attachment of amino-terminated silane. Furthermore, in the N 1s spectra, there was a broad signal detected at ∼400 eV that was not also observed on the reference OH–Si surface, indicative of C–NH_2_ bonding. In Table 1, the relatively small contact angle hysteresis found on the NH_2_–Si surface suggests a homogeneous, dense, and well-ordered monolayer. The monolayer thickness measured on this surface also showed good agreement with the calculated theoretical value (Table 1).

Figure 1 indicates that there was a significant increase in both the C 1s and F 1s peak intensities between the NH_2_–Si and Diaz–Si surfaces. This was expected as a result of the addition of C and F atoms to the surface via attachment of NHS-diazirine. Furthermore, the N 1s spectra for the Diaz–Si, PPh_2_–Si, and PPh_2_–Si (no UV) surfaces all exhibited a shift toward 399.4 eV, which is suggestive of amide bonding [N–(C=O)–C].^50^ In the F 1s spectra for the Diaz–Si, PPh_2_–Si, and PPh_2_–Si (no UV) surfaces, the peak centered around 687 eV indicates the incorporation of −CF_3_ species^50^ present in the attached diazirine moiety. Notably, the F 1s/N 1s XPS signal ratios indicate that there were approximately 4 times more N atoms than F atoms on each surface. This is an indication that the surface concentration of the diazirine groups was ∼10–12 times lower than that of the primary silane layer. This lower degree of coverage is likely attributed to the molecule size and symmetry difference between NHS-diazirine and silane molecules. Nevertheless, goniometry and ellipsometry analysis in Table 1 both indicate an increase in the hydrophobicity and thickness of the Diaz–Si surface relative to NH_2_–Si, which was most likely due to the bulkier headgroups of the attached diazirine moiety. Overall, this evidence suggests covalent amide bond attachment of the carbene precursor into the amino-terminated surface.

The Diaz–Si surface next reacted with DPP under UV light to evaluate the degree of DPP attachment onto the bilayer system. The P 2p core-level spectra shown in Figure 1 demonstrates an increase in signal peak intensity on PPh_2_–Si that was not observed on the other surfaces. Specifically, there was a component at 133 eV, which is indicative of the presence of C–P groups.^50−53^ Furthermore, the only appreciable P 2p/Si 2p ratio was measured from PPh_2_–Si following the photochemical deposition of DPP with UV exposure. This suggests the conversion of the diazirine headgroup to carbene and subsequent reaction with DPP. Table 1 shows that the contact angle of the PPh_2_-Si surface also decreased relative to Diaz–Si possibly as a result of the more polarizable nature of the immobilized P atoms and phenyl rings. The SE thickness measurement also increased after DPP and UV exposure. These observations are evidence of bonding between the phosphine group and the carbene-activated surface.

**Figure 2:**
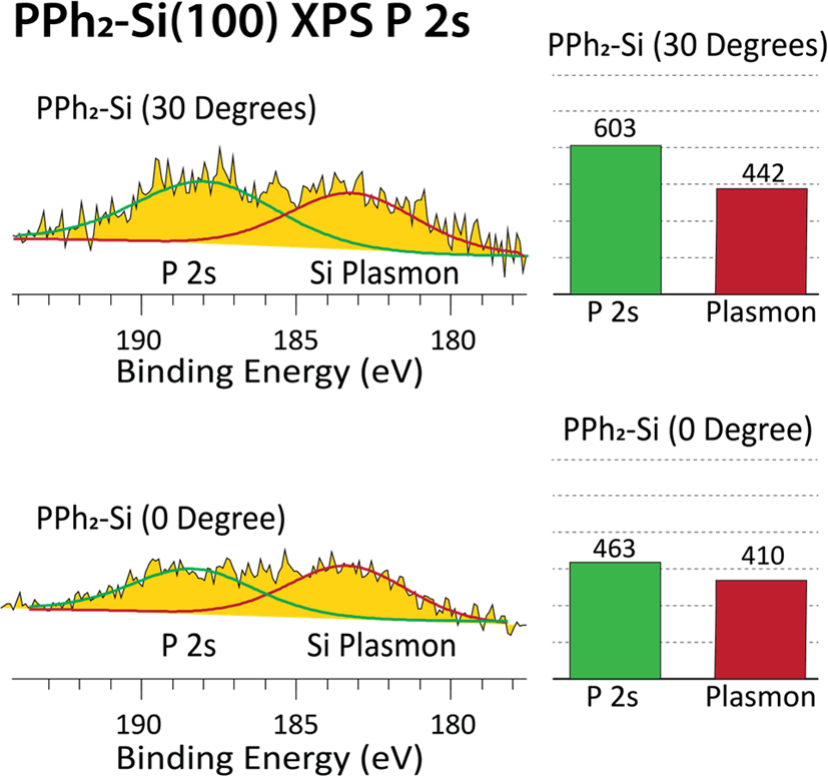
(Left) XPS P 2s core-level XPS spectra for (top) PPh_2_–Si surface with electron detection carried out at an angle of 30° and (bottom) PPh_2_–Si surface with electron detection carried out perpendicular from the surface and (right) histograms showing the integrated area (in arbitrary units) of each respective P 2s and Si plasmon peak.

It should also be noted that, when analyzing the emission of electrons from only the topmost portion of the PPh_2_–Si film (by varying the XPS detection angle), there was an increase in the peak area of the P 2s signal shown in Figure 2, which indicated that the P atoms were primarily bonded atop the Si interface and not distributed throughout the entire XPS analytical depth. Additionally, the ratio of P 2s to Si plasmon signals increased, showing that, as the detection angle became shallower, there were less photoemitted electrons from the Si bulk and more from the top layer of P atoms.

Finally, a control sample bearing the immobilized carbene precursor was also exposed to DPP in a similar manner but without UV light exposure during deposition. This was to test the necessity of UV exposure for carbene generation and phosphine surface bonding. Figure 1 demonstrates the negligible P 2p signal detected on this control surface, and in Table 1, there was strong agreement in goniometry and ellipsometry measurements between the control and Diaz–Si samples. This all suggests that UV radiation was a requirement for P atom surface capture. Overall, the strong agreement between the measured and theoretical thicknesses for each layer shown in Table 1 validates the attachment of each chemical component and is highly suggestive of single monolayers.

It should be noted that the functionalization strategy presented here will most likely require further refinement before implementation in ultrashallow doping applications as a result of limitations of the carbene sublayers. As such, our approach employs a dense sublayer of organic molecules attached to an oxidized silicon. Although similar systems were used in the past for ultrashallow doping,^54^ (1) the underlying oxide interface may limit diffusion of P atoms to Si and (2) C–P and Si–P defects may form (e.g., large diffusion coefficient of C in Si) and induce electrical deactivation of Si.^55^ For example, O’Connell et al. demonstrated that, when annealing a P-modified self-assembled monolayer (SAM) into Si (∼100:1 C/P atomic ratio), up to 20% of the P dopant species was deactivated as a result of C contamination.^56^ Although the molecular system that was used in this study was much smaller (∼24:1 C/P), further reduction in the concentration of C atoms (or F atoms) at the Si interface may be achieved by exploring alternative diazirine or diazo-based compounds with shorter organic linkers.

### Photoreactive Microcontact Printing of the P Dopant onto Functionalized Si(100)

**Figure 3:**
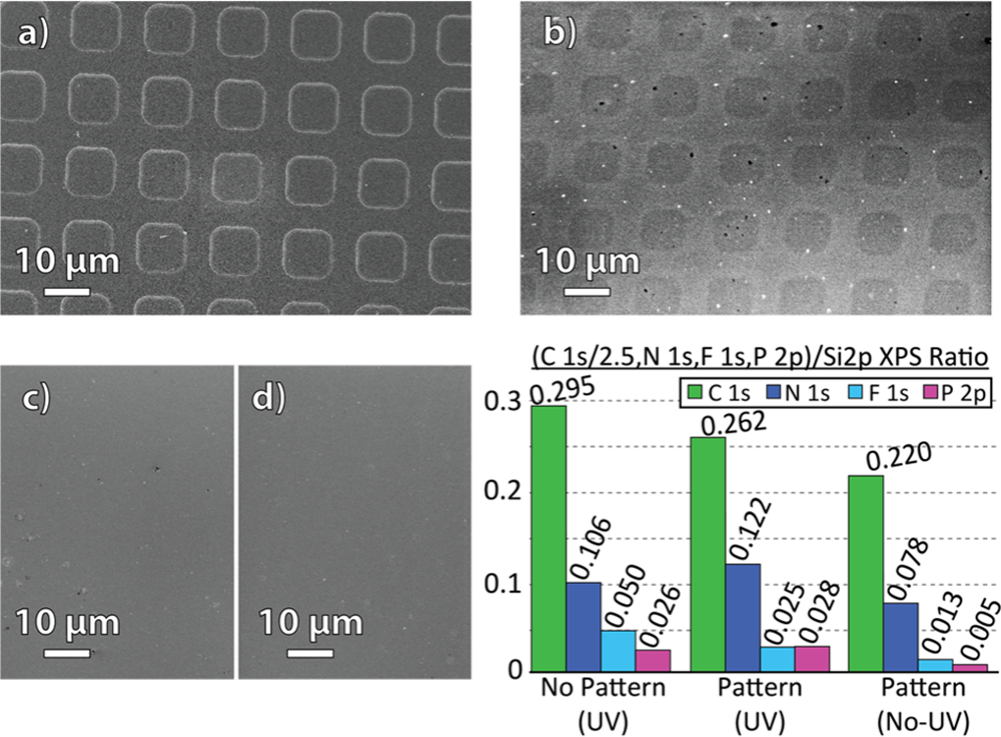
SEM images of the (a) PUA stamp mold bearing 8 μm squares, (b) corresponding P-doped Si(100) surface post-printing, (c) Diaz–Si surface post-printing with DPP-inked stamp and no UV exposure, and (d) Diaz–Si surface post-printing with an inkless stamp and UV exposure and (bottom right) histogram showing XPS ratios of C 1s, N 1s, F 1s, and P 2p over Si 2p electron signals (corrected by the atomic sensitivity factors) on PPh_2_–Si, stamped PPh_2_–Si, and stamped PPh_2_–Si (no UV) substrates.

Microcontact printing is a parallel and scalable technique^57^ for the patterning of small molecules onto inorganic substrates that avoids light diffraction limitations of i-line and UV photolithographic techniques. Its resolution is primarily limited by the lateral diffusivity of the printed molecules and the mechanical deformations of the elastomeric stamps. Here, the previously validated carbene-based functionalization scheme was integrated into a microcontact printing method to orthogonally pattern DPP molecules onto the reactive Si(100) interface. To facilitate this the reactive layer on the substrate must be sufficiently stable to enable efficient coupling of DPP to the activated carbene species. As such, the functionalization approach in this study exploited the stability and order imposed by both the organosilane and carbene precursor compounds to form homogeneously oriented monolayers, which could withstand both stamp contact and removal during attachment of DPP. For a stamping material, PUA was selected as a result of its good moldability, low roughness, and surface energy compatibility with the polarity of the DPP molecule.^45^ This was important because the DPP ink needed to be completely wet and uniformly distributed over the polymer surface to facilitate defect-free complete transfer. Additionally, PUA as an UV-transparent material would enable the activation of the DPP-trapping mechanism of the underlying carbene-terminated substrate. Gas-phase carbene generation and subsequent X–H bond insertion have fast kinetics,^58−60^ which can help facilitate a high rate of pattern transfer in actual applications. The PUA stamp was prepared bearing 8 μm squares to enable site-specific immobilization of phosphines on the receiver surface. The stamp was inked with DPP and then placed in conformal contact with a Diaz–Si surface prior to UV light exposure. After placement of the stamp atop the Diaz–Si substrate, no further force was applied to the stamp/substrate system to avoid the potential for force-induced diffusion and smearing of the DPP molecules. SEM and XPS characterization of the resulting surface after photoreactive contact printing is shown in Figure 3.

The dimensions and orientation of the resulting surface features shown in Figure 3 displayed good agreement with the stamp pattern, indicating effective transfer. To examine whether the change in contrast observed between the 8 μm squares and the background substrate was the result of P coupling or due to the transfer of PUA material to the Diaz–Si surface, a control sample (following the same functionalization and stamping protocol) was made using an inkless stamp under UV exposure. The resulting SEM in Figure 3 showed minimal surface change. The change in contrast observed between the 8 μm squares and the background substrate is attributed to the higher emission of secondary electrons from the heavier P atoms.^61^ Another control sample with the same functionalization and stamping protocol was performed but without the presence of UV light. SEM imaging shown in Figure 3 revealed no apparent pattern transfer, further evidence of stamping selectivity in this photoreactive contact printing process. This approach to patterning small molecular films was demonstrated at the micrometer resolution. We believe that, by adjustment of the ink system and the mechanism of the contact printing activation, it will be possible to further increase the resolution to sub-100 nm dimensions. For example, in the past, we and others have demonstrated diffusionless, sub-50 nm resolution microcontact printing with PUA stamps.^16,37,45,62−64^

The quantitative XPS results in Figure 3 demonstrate that both the homogeneous PPh_2_–Si surface and the stamped PPh_2_–Si surface closely resembled each other chemically, with similar P 2p signals. There was less than a 25% difference in C 1s, N 1s, and P 2p XPS signal ratios between these two surfaces, which suggests that the microcontact printing technique effectively transferred the DPP molecule without degrading the underlying substrate. It also demonstrates that UV radiation was able to activate the surface carbene groups through the PUA stamp. This was key because it was also shown in Figure 3 that the microcontact printing technique without UV light exposure resulted in a near-zero P 2p signal, despite exhibiting C 1s, N 1s, and F 1s ratios indicative of previous functionalization with the diazirine precursor. The slight reduction in the organic signal on this control sample was likely due to partial hydrolysis of the silane–diazirine linker. It should also be noted that the P 2p signal from the stamped PPh_2_–Si surface was measured from a 400 × 400 μm portion of the surface, which included both DPP-functionalized and unmodified regions (i.e., stamped and unstamped) of the surface, and yet still resulted in approximately the same P surface concentration as the homogeneous PPh_2_–Si surface. Therefore, if corrected for the pattern density of the DPP-modified features (25% of the total analytical area), the overall P 2p signal could be about 4 times higher in the DPP regions generated via printing. Similar results have been observed in other studies where reactive contact printing has demonstrated a higher reaction yield than that of non-contact solution phase chemistry.^42^ Lastly, collecting spatially resolved XPS imaging for either P 2p or P 2s electrons would be helpful to further demonstrate the transfer of P atoms exclusively onto the stamped interfaces; however, as a result of the direct overlap of both these signals with strong Si plasmons, it is challenging.

## Conclusion

Semiconductor device manufacturers are facing challenges associated with continuous downsizing of critical dimensions of device components and will continue to do so according to Moore’s law.^62^ Thus, researchers are now searching for novel and efficient processes for the functionalization and patterning of silicon with active molecular or atomic components, such as inorganic-based monomolecular dopant layers. In this study, we have demonstrated an effective method for the micropatterning of P-containing monolayer dopant precursors onto Si(100). This protocol relied upon a bilayer molecular system consisting of a dense amino-silane sublayer and a photoreactive NHS-diazirine overlayer, which is then activated via UV exposure to chemically trap a phosphine-based molecule. This molecular system was subsequently patterned utilizing photoreactive contact printing of DPP under UV light exposure and without mechanical force. The resulting surface patterns matched the surface of the stamp, displaying the high feature uniformity of the printing technique. Ultimately, scalable approaches to patterning and doping small molecular systems, such as the microcontact printing technique implemented in this study, can potentially facilitate a higher resolution and less energy-intensive alternative to traditional top-down micromachining techniques for the continued refinement of templating, ultrashallow doping, and resist-based deposition processes.

## Abbreviations Used

ARXPS: angle-resolved X-ray photoelectron spectroscopy
XPS: X-ray photoelectron spectroscopy
SE: spectroscopic ellipsometry
SEM: scanning electron microscopy
SAM: self-assembled monolayer
